# Optimization of treatment workflow for 0.35T MR‐Linac system

**DOI:** 10.1002/acm2.14393

**Published:** 2024-05-14

**Authors:** Mojtaba Behzadipour, Jatinder Palta, Tianjun Ma, Lulin Yuan, Siyong Kim, Suzanne Kirby, Laurel Torkelson, James Baker, Tammy Koenig, Mateb Al Khalifa, Robert Hawranko, Dylan Richeson, Emma Fields, Elisabeth Weiss, William Y. Song

**Affiliations:** ^1^ Department of Radiation Oncology Virginia Commonwealth University Richmond Virginia USA

**Keywords:** CTgRT, FMEA, MRgRT, MR‐Linac, TDABC

## Abstract

**Purpose:**

This study presents a novel and comprehensive framework for evaluating magnetic resonance guided radiotherapy (MRgRT) workflow by integrating the Failure Modes and Effects Analysis (FMEA) approach with Time‐Driven Activity‐Based Costing (TDABC). We assess the workflow for safety, quality, and economic implications, providing a holistic understanding of the MRgRT implementation. The aim is to offer valuable insights to healthcare practitioners and administrators, facilitating informed decision‐making regarding the 0.35T MRIdian MR‐Linac system's clinical workflow.

**Methods:**

For FMEA, a multidisciplinary team followed the TG‐100 methodology to assess the MRgRT workflow's potential failure modes. Following the mitigation of primary failure modes and workflow optimization, a treatment process was established for TDABC analysis. The TDABC was applied to both MRgRT and computed tomography guided RT (CTgRT) for typical five‐fraction stereotactic body RT (SBRT) treatments, assessing total workflow and costs associated between the two treatment workflows.

**Results:**

A total of 279 failure modes were identified, with 31 categorized as high‐risk, 55 as medium‐risk, and the rest as low‐risk. The top 20% risk priority numbers (RPN) were determined for each radiation oncology care team member. Total MRgRT and CTgRT costs were assessed. Implementing technological advancements, such as real‐time multi leaf collimator (MLC) tracking with volumetric modulated arc therapy (VMAT), auto‐segmentation, and increasing the Linac dose rate, led to significant cost savings for MRgRT.

**Conclusion:**

In this study, we integrated FMEA with TDABC to comprehensively evaluate the workflow and the associated costs of MRgRT compared to conventional CTgRT for five‐fraction SBRT treatments. FMEA analysis identified critical failure modes, offering insights to enhance patient safety. TDABC analysis revealed that while MRgRT provides unique advantages, it may involve higher costs. Our findings underscore the importance of exploring cost‐effective strategies and key technological advancements to ensure the widespread adoption and financial sustainability of MRgRT in clinical practice.

## 1 INTRODUCTION

Magnetic resonance guided radiation therapy (MRgRT) is a cutting‐edge radiation therapy technique that uses real‐time magnetic resonance imaging (MRI) to guide and adapt radiation delivery in response to changes in tumor position, shape, and size during treatment. By using MRI, MRgRT can provide high‐resolution imaging of the tumor and surrounding healthy tissues, allowing for accurate targeting of the tumor and sparing of healthy tissues.^1^ MRgRT provides functional information and superior soft‐tissue visualization, allowing clinicians to accurately target the tumor.^2^ This is particularly important for tumors located in areas of the body that are not easily visible under computed tomography (CT) scans, such as the brain, spine, abdomen, and pelvis.^3^, ^4^ Another key advantage of MRgRT is its ability to track tumor motion in real‐time during treatment. This is especially important for tumors located in organs that move due to breathing, such as liver, lung, and pancreas.^5^

Due to its complexity and novelty, MRgRT entails new forms of hazards, such as those connected with the integration of MR imaging into radiotherapy equipment, risks due to utilizing MR images for planning rather than CT images, etc.^6^ The workflow for treatment on the 0.35T MRIdian MR‐Linac system (ViewRay, Inc.) involves a collaborative effort from various members of the clinical team. Given the complexity and diversity of tasks involved, it becomes essential to assess the workflow for potential failure modes. The Task Group (TG)‐100 report provides a framework for improving the risk‐assessed quality and safety of both new and old technologies and procedures, including the application of failure modes and effects analysis (FMEA) to identify and mitigate potential hazards and risks associated with radiation therapy treatments.^7^ In this study, we applied TG‐100 guidelines to identify high‐risk failure modes, their associated effects on treatment quality and patient safety, and possible root causes for the 0.35T MRIdian MR‐Linac system workflow.

Given the complexities inherent in the current workflow and the potential risks and hazards it presents, a thorough and rigorous assessment of the associated costs becomes indispensable. Moreover, should any modifications be contemplated to mitigate the identified hazards, a meticulous reevaluation of the costs becomes imperative to ensure the sustainability and viability of care delivery expenses. Time‐driven activity‐based costing (TDABC) is a method that can be used to evaluate the cost of MRgRT treatment compared to traditional radiation therapy. It was first introduced in 2004 by Robert S. Kaplan and Steven R. Anderson and was developed as a response to the limitations of traditional cost accounting methods, which often fail to accurately capture the true cost of healthcare processes. TDABC involves tracking the time and resources (i.e., personnel, space, equipment, and materials) used during each step of a healthcare process and assigning a cost to each resource based on its unit cost.^8^ This enables healthcare providers to understand the true cost of each process and identify areas where costs can be reduced.

In this study, we integrate the FMEA approach with TDABC, providing a novel and comprehensive framework for MRgRT evaluation. We not only assess the workflow in terms of safety and quality, as done through FMEA, but also account for the economic implications using TDABC, providing a holistic understanding of all aspects of the treatment workflow. In light of ViewRay's recent bankruptcy, the financial viability of MR‐guided linacs, warrants critical examination. This event underscores the importance of our study, which aims to provide a comprehensive workflow assessment of the MRIdian system, including an in‐depth cost analysis. Our research is particularly relevant as it elucidates the inherent higher costs associated with common features of MR‐guided linacs. Notably, all MR‐guided linacs are designed to facilitate beam gating, a capability that significantly extends treatment time and, consequently, the overall treatment costs. Five‐fraction liver, lung, and pancreatic stereotactic body radiation therapy (SBRT) treatments with the MRIdian system were selected to compare with those on the TrueBeam Linac system (Varian, Palo Alto, California, USA) in our clinic. By applying TDABC principles, we were able to assess the cost implications of MRgRT and gain insights into potential areas for cost optimization. Through this novel analysis, the combination of TDABC and FMEA enables financial and logistic workflow optimization for online MR‐guided adaptive radiotherapy (ART).

## 2 METHODS

### 2.1 Failure modes and effects analysis (FMEA)

The FMEA process examined the entire treatment course, encompassing various stages starting with patient MR‐appropriateness assessment and record review and ending with the last treatment fraction and documentation.

**FIGURE 1:**
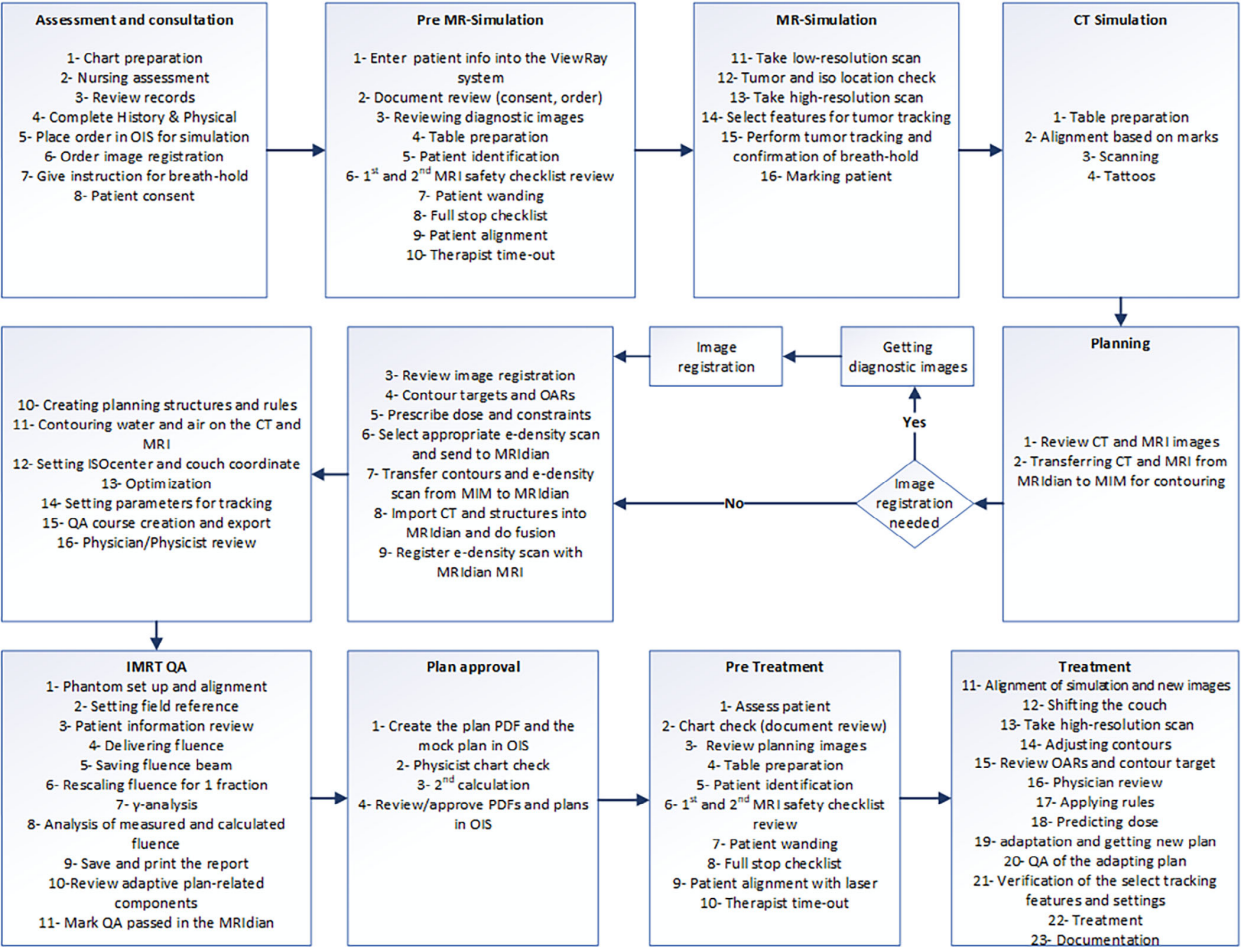
Process map of the MRIdian MR‐Linac SBRT treatment workflow in our department. iso, isocenter; OIS, oncology information system (we use ARIA treatment management system in our department).

A multidisciplinary team was formed with the objective of creating a process map to codify various steps within the workflow. The primary goal was to identify potential failure modes and their corresponding causes and effects. The process evaluation team comprised three radiation therapists, three medical physicists, three medical physics residents, two attending physicians, and two dosimetrists to follow the TG‐100 methodology. The team members assigned to each stage of the treatment workflow engaged in detailed discussions to identify all sub‐steps. The inputs from the radiation oncology care team members were utilized to create a workflow map (Figure 1).

The MRIdian daily adaptive workflow map illustrates the detailed steps involved in the treatment process. It begins with the initial assessment and consultation, where the radiation oncologist evaluates the patient's condition to determine the appropriate course of treatment. This stage is crucial for understanding the patient's needs and developing a personalized treatment plan. The next step is a 0.35T MRIdian MRI simulation, where the patient undergoes MR imaging to obtain detailed images of the tumor and surrounding tissues. This step also involves assessing the trackability of the real‐time MRI cine images and determining whether a visible feature can be reliably identified by the tracking algorithm. Additionally, within this step, the patient's position and setup are documented. These images provide essential information for accurate target localization and treatment planning. Following an MRI simulation, a CT simulation takes place. This involves performing CT scans to gather electron density data. The combination of MR and CT images allows for precise delineation of the tumor and critical structures. This step of the process ensures geometric fidelity and allows for dose calculations. Once the imaging data are obtained, the planning stage begins. Dosimetrist, using the MR and CT, creates a treatment plan using inverse optimization. This involves determining the optimal radiation dose distribution, using a set of available beam angles (typically 20−30), with step‐and‐shoot intensity‐modulated radiotherapy (IMRT) delivery enabled by a unique dual‐stack double‐focused multi‐leaf collimator (MLC) design.^9^, ^10^ Currently, a volumetric‐modulated arc therapy (VMAT) delivery mode is not available on the MRIdian MR‐Linac system. Before treatment can commence, the plan undergoes rigorous patient‐specific quality assurance (QA) & quality control (QC) checks conducted by a medical physicist. This thorough evaluation ensures that the delivered radiation adheres to the intended plan and meets the required standards. The treatment phase relies heavily on the radiation therapists, who play a vital role in patient setup, treatment delivery, and treatment monitoring. Radiation therapists employ their expertise to provide continuous oversight and monitor the patient during the treatment. Throughout the treatment process, radiation oncologists have overall responsibility of managing patient's radiation treatment. They regularly evaluate the patients’ progress, review medical imaging data, and direct online adaptive replanning as necessary. Their expertise in target delineation, dose prescription, organ segmentation, and overall plan evaluation ensures that the MRgRT is delivered precisely and accurately.

According to the process map, team members listed detailed sub‐steps and their corresponding failure modes based on their own clinical experience. After the identification of potential failure modes, the team proceeded to assess the occurrence (O), severity (S), and detectability (D) of each failure mode based on the TG‐100 scoring criteria.

**FIGURE 2:**
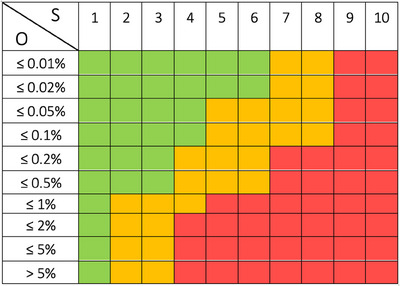
Risk matrix compatible with TG‐100 scoring criteria. Colors show risk levels: low risk (green), medium risk (yellow), and high risk (red).

Using this information, the team assigned a risk priority number (RPN = O×S×D) to prioritize the failure modes. In accordance with the TG‐100 methodology, the team identified the top 20% RPNs and the top 20% severity. To further enhance the risk analysis process, the team introduced a minor modification to the TG‐100 approach while ensuring adherence to its scoring criteria for the purpose of classifying risk levels, as presented in Figure 2. This risk matrix facilitated the classification of failure modes into distinct risk levels, considering both their severity and occurrence ratings: high risk (red), medium risk (yellow), and low risk (green). Following the TG‐100 methodology, high‐severity failure modes (S = 9–10) were categorized as high risk, regardless of their occurrence probability, as they require mitigation measures to ensure patient safety. To obtain consensus‐based scoring, two different approaches were employed. Medical physicists and medical physics residents independently assigned scores, and the resulting averages were used. Meanwhile, other members of the clinical team were engaged in continuous comprehensive discussions regarding each failure mode, ultimately arriving at mutually agreed upon scores. After identifying and addressing the top‐priority failure modes and optimizing the workflow in terms of safety through educational and training initiatives, a resource distribution process map was set up for TDABC analysis.

### 2.2 Time‐driven activity‐based costing (TDABC)

Once the workflow was documented, a TDABC analysis was performed to assess the costs and compare them with the conventional cone‐beam CT‐guided RT (CTgRT) implemented in our clinic. A TDABC analysis consists of seven steps outlined by Kaplan and Porter ^8^: (1) selecting the treatment or service, 2) defining the care delivery value chain (CDVC), (3) creating a detailed process map, (4) estimating time, (5) estimating resource costs, (6) calculating the capacity cost rate (CCR), and (7) determining total costs. A TDABC analysis was performed to assess the costs and compare MRgRT and CTgRT treatment workflows for administering a typical five‐fraction SBRT to patients with liver, lung, and pancreatic cancers.

For the MRgRT workflow, our institution implemented the 0.35T MRIdian MR‐Linac system that combines 0.35T MRI with a 6MV flattening filter free (FFF) beam linear accelerator. The treatment plan is developed using the MRIdian treatment planning system (TPS). Throughout the course of treatment, the patient is closely monitored by the radiation therapy team. This may involve regular on‐treatment visits (OTVs) to ensure that the treatment is proceeding according to the plan and that any side effects are being effectively managed. After treatment is complete, the patient undergoes follow‐up appointments to monitor treatment outcome and associated acute and late toxicities, if any.

We use TrueBeam linear accelerator (Varian, Palo Alto, California, USA) equipped with on‐board cone beam CT (CBCT) for CTgRT. Before treatment begins, patients undergo a CT simulation to determine the exact location and shape of the tumor and normal tissues. This information is used to develop a VMAT (RapidArc) plan using Varian's Eclipse TPS. Radiation oncologist then reviews and approves the treatment plan, ensuring that the radiation dose distribution is optimal for given geometry. Similar to the MRgRT, after the treatment plan is finalized, it undergoes a patient‐specific QA process. When the plan passes the QA, patient is ready to begin the course of treatment. During treatment, patients are immobilized using the Orfit SBRT solution. In addition, the TrueBeam Linac at our institution is equipped with the Identify surface imaging system, which allows for real‐time monitoring of the patient's surface position and patient motion during the treatment.

The fundamental differences between the two workflows are in their approaches to motion management and adaptive strategies. For MRgRT, motion management is accomplished through breath‐hold techniques, real‐time target tracking, and beam gating. In contrast, CTgRT in our practice does not employ real‐time target tracking, but with surface motion monitoring. 4D motion management is achieved through the creation of Internal Target Volume (ITV), and it may involve the use of breath‐hold, compression belts, or shallow kinetics induced by the metronome (SKIM) strategy as well.^11^ The other fundamental distinction pertains to MRgRT's capability for online adaptive replanning, a feature that is currently absent in our TrueBeam platform based CTgRT.

**FIGURE 3:**
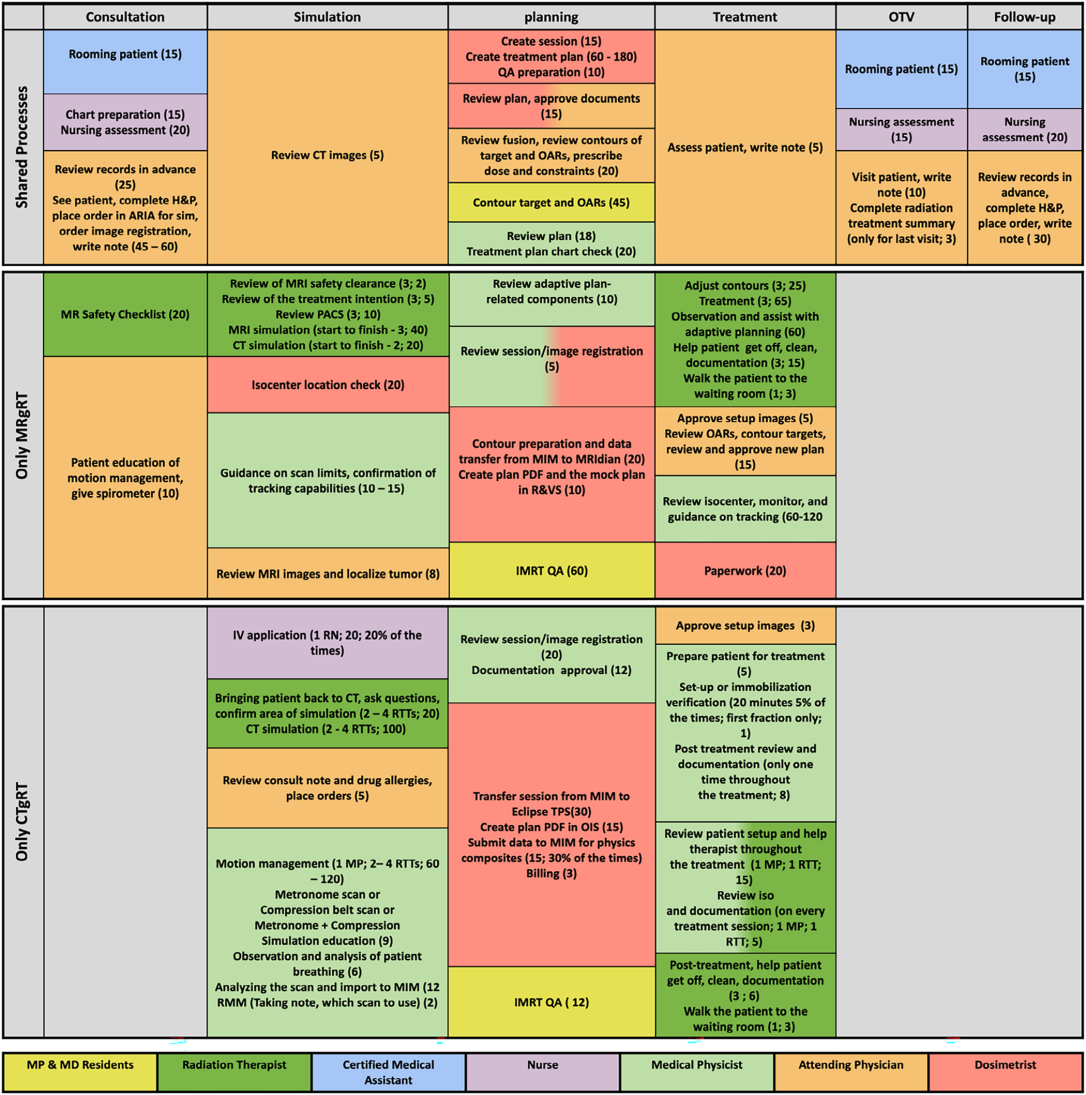
Process maps for CTgRT and MRgRT workflows (steps are not ordered in time). Colors in each box represent the responsible team member, with multiple colors indicating joint responsibilities. The numbers in parentheses show the time required for that task in minutes and percentage of occurrence possibility if included, as those tasks are not applicable to every patient. Regarding radiation therapists, the first number is the number of staff involved in that step. MP, medical physicist; RTT, radiation therapist.

A resource distribution process map, including the steps involved in both CTgRT and MRgRT workflows, was developed based on the input of each team member (Figure 3). These stages included consultation, simulation, planning, the actual delivery of treatment over five SBRT fractions, an OTV, and one follow‐up visit. Each stage consists of detailed steps, the types of personnel involved in that specific step, and the time required for each individual step. The time estimation for all steps was obtained through in‐person interviews with the radiation oncology care team members involved in those steps.

Personnel costs include the salaries, as well as any overhead costs associated with their employment, such as benefits, bonuses, etc. Information on the compensation of staff working in the Department of Radiation Oncology was obtained by the institution's human resources department. The salaries and benefits for each personnel category were based on the average compensation for that role within the institution. Personnel were required to adhere to a standardized annual working schedule of 2080 h. Equipment and space useful lives were assumed 10 and 25 years, respectively. The availability of spaces and equipment is 10 h per day, encompassing a span of 260 days within a year. However, an exception applies to the PET/CT simulator and its accompanying space, as their availability is limited to 8 h per day. Regarding the equipment, CCRs were calculated based on their sales price and recorded maintenance costs. No disposable materials were used in either MRgRT or CTgRT. The CCR for different resources (personnel, equipment, and space costs) were then calculated by their annual costs divided by their working hours per year (see Table 1). CCRs were then multiplied by the time estimates [min] for each step in the process map, resulting in the total cost for that specific step and summed over all the steps to derive the total cost for each modality.

**TABLE 1:** Personnel, equipment, and space CCRs.

Personnel	Annual working days	Daily working hours	CCR ($/min)
Radiation therapist	260	8	$0.71
Certified medical assistant	260	8	$0.32
Nurse	260	8	$0.64
Medical physicist	260	8	$2.34
Attending physician	260	8	$4.40
Dosimetrist	260	8	$1.14
MP & MD residents	260	8	$0.59
**Space (Vault)**	**Useful life (year)**	**Daily usage (h)**	**CCR ($/min)**
TrueBeam	25	10	$0.31
MRIdian	25	10	$0.36
PET/CT	25	8	$0.08
**Equipment**	**Useful life (year)**	**Daily usage (h)**	**CCR ($/min)**
TrueBeam	10	10	$4.04
MRIdian	10	10	$8.01
Cannon PET/CT Simulator	10	8	$3.21
Identify	10	10	$0.47
Orfit SBRT solution	10	10	$0.05

## 3 RESULTS

A total of 279 failure modes were identified, out of which 30 were classified as high‐risk failure modes, 55 were classified as medium‐risk failure modes, and the remaining failure modes were categorized as low‐risk. Among these, 52 failure modes were identified as the top 20% based on their RPNs. The top 20% RPNs were separately identified for each type of personnel.

The compiled results, highlighting the ranked list of failure modes based on their RPNs, can be found in Table 2. The last two columns in the table provide essential information, indicating whether a failure mode falls within the top 20% in terms of severity and specifying its corresponding risk level.

**TABLE 2:** List of high‐priority failure modes (top 20% RPNs).

Process step	Top 20% RPN failure modes	Potential cause of failure	Potential effects of the failure	O	S	D	RPN	Top 20% severity	Risk level
Dosimetrist: Setting parameters to do adaptive planning and tracking	Selecting the wrong prescription templates	Suboptimal MRIdian tools for management of templates with current software version	Critical constraints can be missed when the wrong or outdated template is selected	7	8	4	224	Yes	
Therapist: Adjusting contours	Not readjusting the ring	Human failure (inattention),	Tissue overdose/underdose	4	8	4	128	Yes	
Therapist and Attending Physician: Adjusting contours	Contouring structures incorrectly	Human failure (inexperience or inattention)	Tissue overdose/underdose	6	7	3	126	Yes	
Therapist: Review OARs and contour target	Not reviewing OARs	Human failure (inattention),	Tissue overdose/underdose	3	8	5	120	Yes	
Therapist: Alignment of sim and new (daily) images	Not checking all OARs	Human failure (inattention)	Normal tissue overdose	3	8	5	120	Yes	
Physics, Dosimetrist, and Physician: Optimization and getting new plan	Proceeding to treatment before checking that dose to OARs did not exceed max dose	Human failure (inattention)	OAR overdose	2	7	7	98	Yes	
Therapist: Applying rules	Not applying rules	Human failure (inattention),	Not achieving Rx dose	3	8	4	96	Yes	
Physics QA: Phantom set up and alignment	Imperfect alignment	Lack of experience, being in rush, being disrupted	Suboptimal dose delivery, phantom misaligned, geometric failure gamma analysis, failing gamma criteria due to poorly aligned phantom	4	5.33	4.33	92.44	No	
Physics QA: Setting field reference	Mistake in setting the correct reference field	Lack of experience, being in rush, being disrupted, misinput/transcribed values, misunderstanding of the MU scaling factor calculation process	Wrong dose, suboptimal plan	2.67	5	6.67	88.89	Yes	
Therapist: Chart check (document review)	Treatment without IMRT QA	Human failure (inattention)	Mistreatment	3	7	4	84	Yes	
Dosimetrist: Creating planning structures and rules	Forget to save the rules	easy click of a button to forget during creation of rules	It may have a big effect on what is treated if not caught at time of adaptation	3	9	3	81	Yes	
Therapist: Enter patient info	Put wrong patient information	Human failure (inattention)	Treat wrong patient	2	10	4	80	Yes	
Attending physician: Review records in advance	Forget radiation sensitizing medical condition	Unusual, not known or documented	Toxicity to patient	2	8	5	80	Yes	
Attending physician: Consultation	Forget to stop radiation sensitizing treatment including chemotherapy, targeted treatment, medications	Outside records	Toxicity to patient	2	8	5	80	Yes	
Therapist: Take low‐ and high‐resolution scan	Therapist not noticing patient did not hold breath for entire length of scan	Human failure (inattention)	Inadequate Tx	5	5	3	75	No	
Physicist: Treatment	Patient movement	Uncontrollable significant motion both external and internal	longer treatment / repeat alignment imaging	4.33	6	2.67	69.33	Yes	
Physics QA: Patient information review	Wrong number of fractions	Not being careful or improperly instructed	Wrong delivered dose	3	7.67	3	69	Yes	
Therapist: Patient alignment	Too far off‐center patient (during treatment)	Human failure (inattention),	Decrease in tumor visibility, fall, injury, Inadequate image	2	8	4	64	Yes	
Dosimetrist: Creating planning structures and rules	Wrong expansion or rule creation	process of creating rules is complex and prone to error, but physics does a thorough review prior to treatment	This could have a big effect on what is treated if not caught at time of adaptation	7	9	1	63	Yes	
Therapist: Adjusting contours	Not contouring all OARs within the ring^a^	Human failure (inexperience or inattention)	Tissue overdose/underdose	5	6	2	60	Yes	
Therapist: Full stop checklist	Forgetting to complete the full stop checklist or only partially filling it out	Being behind schedule, rush	Projectile/patient harm/burning/injury	3	6	3	54	Yes	
Physicist: Review adaptive plan‐related components	Forgetting to verify ruls^b^	Significant anatomical variations	Sub‐optimal dose distribution being delivered to the patient	3.67	5.33	2.67	52.33	Yes	
Physicist: Perform tumor tracking and confirmation of breath‐hold	Tumor not being tracked correctly	Sub‐optimal image quality	longer treatment, partial treatment, unable to treat patient, re‐simulation of the patient	3.67	4.67	3	51.13	Yes	
Therapist: Patient alignment	Forgetting to give the squeeze ball to the patient	Human failure (inattention)	Inadequate patient communication	6	8	1	48	Yes	
Therapist: Applying rules	Not applying all the rules	Human failure (inattention)	Not achieving Rx dose	2	8	3	48	Yes	
Therapist: Applying rules	Not verifying that all the rules were correct	Human failure (inattention)	Not achieving Rx dose	3	8	2	48	Yes	
Therapist: Treatment Documentation	Forget EOT paperwork	Human failure (inattention)	Might delay a fraction	4	4	3	48	No	
Therapist: Patient alignment	Nonreproducible positioning	Human failure (inattention)	Extra set‐up time	5	3	3	45	Yes	
Therapist: Full stop checklist	Entering the zone 4 with ferrous material (patient, equipment, staff, etc.)	Forgetting safety checks	Projectile/injury	2	7	3	42	Yes	
Physics QA: Setting field reference	Mistake in setting the correct MU	Misunderstanding of the MU scaling factor calculation process, Lack of training, not following SOP workflow	Computing an incorrect MU scaling factor which will unfairly compare the delivered and prepared dose distributions, wrong dose, suboptimal plan	2.67	5.67	2.67	40.3	No	
Attending physician: Review records in advance	Forget previous radiation treatment	physician not asking about previous RT	Overdose	1	8	5	40	No	
Therapist: Verification of the select tracking features and settings	Wrong confidence value	Human failure (inattention)	Overdose or underdose	2	5	4	40	No	
Therapist: Patient alignment	Improper positioning of the headphone on patient's ear	Patient not communicating with RT	Damage to patient's eardrum	2	3	6	36	No	
Therapist: Patient alignment	Not properly aligned on coils	Human failure (inattention)	Poor imaging quality	6	3	2	36	Yes	
Therapist: Verification of the select tracking features and settings	Tracking structure not within SI lines	Human failure (inattention)	Inaccurate tracking	2	3	6	36	No	
Therapist: Treatment Documentation	Not converting calendar back to original per physician preference	Human failure (inattention)	Longer treatment time	3	6	2	36	No	
Physics QA: Delivering the fluence	Selecting the wrong IMRT QA plan (in case of multiple available IMRT QA plans)	Not being careful, error in the spreadsheet, improperly instructed, not following the IMRT QA procedure, plans not labeled clearly, not reviewing history of the patient	Gamma analysis failure, QA failure, delivering the wrong fluence, re‐analysis of QA	3	8.67	1.33	34.67	Yes	
Physics QA: Analysis of the measured and the calculated fluence	Delivering the wrong IMRT QA plan	Being distracted, not being careful, confusion (when patient has multiple IMRT QA plans), Plans not labeled clearly	QA failure, Wrong dose and dose distribution	3	8.67	1.33	34.67	Yes	
Attending physician: Adaptive treatment planning	Incorrect target or OAR contouring or OAR review including incomplete contours	Covering doctor, unfamiliar therapist, difficult anatomy	Overdose	2	8	2	32	Yes	
Therapist: Patient alignment	Too off‐center patient (during simulation)	Patient not mobile/hard to move	Decreased tumor visibility, fall, injury	2	8	2	32	Yes	
Therapist: Verification of the select tracking features and settings	Wrong frames per second	Human failure (inattention)	Failure of structure tracking	2	4	4	32	Yes	
Dosimetrist: Contouring air on the CT and MRI	Missing air on either scan or overdrawing it	Excessive amount of air pockets to contour manually	overall change to plan is small when a few pockets of air are not accounted for	4	4	2	32	Yes	
Dosimetrist: Setting parameters to do adaptive planning and tracking	Forgetting to avoid arms and rounded edges of the table	Dosimetrist doesn't predict accurately which beams angles to avoid arms and table edge	Undesirable dose to arms or slightly inaccurate plan due to treating through angled portion of table edge	3	5	2	30	Yes	
Attending physician: Review records in advance	Forget radiation sensitizing treatment (medication, chemotherapy, targeted treatment)	Outside records	Toxicity to patient	1	8	2	16	Yes	
Attending physician: Planning	Contour wrong OAR or incomplete OAR	Confusion due to different modalities and time points of imaging studies in the system.	Toxicity	2	8	1	16	No	
Therapist: Patient alignment	Squeeze ball not plugged in	Human failure (inattention)	Inadequate patient communication	2	8	1	16	Yes	
Attending physician: Adaptive treatment planning	Overlooked dose hot spots outside the recontouring ring	Covering doctor, unfamiliar therapist, difficult anatomy	Toxicity	2	3	2	12	No	

^a^ The “ring” refers to a 3 cm uniform expansion axially and 2 cm expansion in superior‐inferior direction. Within this defined boundary, OARs are subject to adaptive modification.

^b^ Rules are pre‐defined scripts used for creating key planning structures such as the combination of OARs, margin expansion, and rings.

Personnel costs for different stages of the treatment and personnel types for MRgRT and CTgRT are shown in Table 3. The total personnel costs for five‐fraction SBRT treatment with MRgRT and CTgRT are $4678.13 and $2770.13, respectively. The total equipment and space costs for MRgRT are $4471.15 and $199.04, respectively. In comparison, CTgRT incurs costs of $1343.97 for equipment and $74.85 for space. Finally, the total costs for five‐fraction SBRT MRgRT and CTgRT are $9348.32 and $4188.95, respectively.

**TABLE 3:** CTgRT (above), MRgRT (middle), and CTgRT‐MRgRT (below) personnel costs for all treatment stages.

CTgRT							
	Consultation	Simulation	Planning	Treatment	On‐treatment visit	Follow‐up	Total
Radiation therapist	$0.00	$255.60	$0.00	$340.80	$0.00	$0.00	$596.40
Certified medical assistant	$4.80	$0.00	$0.00	$0.00	$4.80	$4.80	$14.40
Nurse	$22.40	$2.56	$0.00	$0.00	$9.60	$12.80	$47.36
Medical physicist	$0.00	$278.46	$163.80	$489.06	$0.00	$0.00	$931.32
Attending Physician	$341.00	$44.00	$154.00	$176.00	$57.20	$132.00	$904.20
Dosimetrist	$0.00	$0.00	$242.82	$0.00	$0.00	$0.00	$242.82
MP & MD Residents	$0.00	$0.00	$33.63	$0.00	$0.00	$0.00	$33.63
Total	$368.20	$580.62	$594.25	$1,005.86	$71.60	$149.60	$2,770.13
MRgRT							
Radiation therapist	$14.20	$149.81	$0.00	$1,150.20	$0.00	$0.00	$1,314.21
Certified medical assistant	$4.80	$0.00	$0.00	$0.00	$4.80	$4.80	$14.40
Nurse	$22.40	$0.00	$0.00	$0.00	$9.60	$12.80	$44.80
Medical physicist	$0.00	$29.25	$124.02	$1,053.00	$0.00	$0.00	$1,206.27
Attending Physician	$385.00	$57.20	$154.00	$550.00	$57.20	$132.00	$1,335.40
Dosimetrist	$0.00	$22.80	$222.30	$456.00	$0.00	$0.00	$701.10
MP & MD Residents	$0.00	$0.00	$61.95	$0.00	$0.00	$0.00	$61.95
Total	$426.40	$259.06	$562.27	$3,209.20	$71.60	$149.60	$4,678.13
CTgRT – MRgRT							
Radiation therapist	‐$14.20	$105.79	$0.00	‐$809.40	$0.00	$0.00	‐$717.81
Certified medical assistant	$0.00	$0.00	$0.00	$0.00	$0.00	$0.00	$0.00
Nurse	$0.00	$2.56	$0.00	$0.00	$0.00	$0.00	$2.56
Medical physicist	$0.00	$249.21	$39.78	‐$563.94	$0.00	$0.00	‐$274.95
Attending Physician	‐$44.00	‐$13.20	$0.00	‐$374.00	$0.00	$0.00	‐$431.20
Dosimetrist	$0.00	‐$22.80	$20.52	‐$456.00	$0.00	$0.00	‐$458.28
MP & MD Residents	$0.00	$0.00	‐$28.32	$0.00	$0.00	$0.00	‐$28.32
Total	‐$58.20	$321.56	$31.98	‐$2,203.34	$0.00	$0.00	‐$1,908.00

To assess the potential time and cost savings associated with various (and reasonably expected) technological advancements for MRgRT, data from the delivery reports of our institution's 40 patients treated for liver, lung, and pancreatic cancers, who underwent five‐fraction SBRT between January 2022 and March 2023, were analyzed.

The recorded data extracted from MRIdian's treatment delivery report includes parameters such as “Total Delivered Monitor Unit (MU),” “Total Delivery Time,” and “*Beam On Time*.” It is important to note that the *Beam On Time* refers to the time during which the MR‐Linac is not only actively delivering radiation but also includes the MLC movements between segments (for step‐and‐shoot IMRT), and the gantry rotation where the radiation is actually stopped. The *Beam On Time* and contouring represent the most substantial time‐intensive phases within the workflow, concurrently harboring the greatest potential for cost reduction.^12^ Contouring not only has cost‐saving benefits but also plays a pivotal role in risk mitigation. For instance, the use of *auto‐segmentation* in contouring can lead to both reduced costs and decreased treatment risks. In this section, we delve deeper into the respective benefits inherent to each of these phases.

### 3.1 Auto segmentation cost savings

Potentially the biggest cost‐saving technology to consider is the introduction of real‐time *auto‐segmentation*, driven by recent exciting developments in artificial intelligence (AI) algorithms.^13^, ^14^, ^15^ By eliminating, the most laborious manual step in the entire online MR guided ART workflow, especially during the time‐critical online re‐planning, the auto‐segmentation technology would enable efficient streamlining of the re‐planning process and potentially significantly decrease personnel, equipment, and space costs. In the MRgRT workflow at our clinic, the manual contouring during planning takes approximately 30−45 min by a radiation oncology resident, while adjusting additional contours by radiation therapists take 20−25 min at each online replanning session. The latter includes not only the personnel costs for three radiation therapists, one medical physicist, and one dosimetrist, but also equipment and space costs. Our analysis demonstrated that integrating auto‐segmentation into the MRIdian adaptive workflow leads to significant cost savings, with personnel costs reduced by $727.80, equipment costs reduced by $1001.60, and space costs reduced by $44.87, resulting in a whopping total cost savings of $1774.27 per SBRT treatment course for MRgRT.

### 3.2 Beam on time cost savings

The difference between the *Beam On Time* and the *Total Delivery Time* comes from accounting for “beam hold” during when target is out of bounds as MRIdian allows real‐time, automated target tracking, and beam gating. This time difference can be minimized through the implementation of a dynamic MLC tracking technology where the MLC segments are dynamically adapted to the moving anatomy/target, which ensures continuous “beam on” during treatment. Our 40‐patient treatment record indicates that such a technology (i.e., dynamic MLC tracking, etc.) can result in a meaningful treatment time reduction of approximately 3.56 min per SBRT fraction. This reduction, in turn, translates to noteworthy cost savings, amounting to $99.86 in personnel costs, $142.63 in equipment costs, and $6.39 in space costs, ultimately culminating in total cost savings of $248.88 per SBRT treatment course. Further, by integrating the hypothetical MLC tracking technology with sliding‐window VMAT delivery, which enables more efficient radiation delivery through both MLC and gantry motions (i.e., less *Beam On Time*), it is possible to achieve even higher time savings. This combination has the potential to reduce the treatment duration by approximately 8.53 min per SBRT fraction. As a result, significant cost savings can be realized, including $339.12 in personnel costs, $484.38 in equipment costs, and $21.70 in space costs. Ultimately, the cumulative effect of the VMAT + MLC Tracking amounts to a total cost reduction of $845.20 per SBRT treatment course for MRgRT.

Another potential approach to further reducing cost is by simply increasing the dose rate of the 6MV FFF beam produced by the MRIdian Linac. Currently, the MRIdian Linac operates at the invariable dose rate of 600 MU/min, which is significantly lower than the typical dose rate of its counterpart, TrueBeam, which runs at 1400 MU/min. By simply increasing the dose rate from 600 to 1400 MU/min, the *Beam On Time* can be significantly shortened. This efficiency improvement can lead to more effective utilization of resources and has the potential to increase patient throughput. Specifically, this upgrade can result in a reduction of personnel costs by $112.76, equipment costs by $161.06, and space costs by $7.22, amounting to a total cost savings of $281.03 per SBRT treatment course for MRgRT.

Synthetic CT, a technology that generates CT‐like electron density data from MR images, presents another potential avenue for cost reduction by eliminating the requirement for a separate CT simulation. However, since the real CT simulation is performed only once during the entire treatment course and does not significantly impact personnel and equipment utilization, its elimination does not contribute significantly to the overall treatment cost. Consequently, the cost savings achieved by removing the real and only‐once CT simulation are relatively modest, amounting to $94.11 per SBRT treatment course for MRgRT.

While the total MRgRT SBRT treatment course takes 1346 min, considering all of these technologies together, the total treatment time could be reduced by 270.55 min (∼20%). Additionally, the implementation of all of these technologies together could reduce the total MRgRT costs to $6353.71 per SBRT treatment course. It is worth noting that while MRgRT may still have higher costs compared to CTgRT ($4188.95) due to the specialized MRI equipment, infrastructure, and unique online ART workflow, the long‐term cost‐effectiveness versus clinical outcome should be evaluated. Potential benefits of MRgRT, such as improved treatment accuracy, reduced toxicity, and better patient outcomes, could offset the initial investment over time.^16^, ^17^, ^18^

## 4 DISCUSSION

The present study employed FMEA in combination with TDABC to assess the workflow and associated costs of MRgRT compared to conventional CTgRT for administering five‐fraction SBRT to patients with liver, lung, and pancreatic cancers in our clinic. Through the FMEA process, a multidisciplinary team meticulously examined the entire treatment course, identifying potential failure modes, their causes, and their effects. The analysis yielded a comprehensive understanding of critical hazards and risks in the radiotherapy workflow, leading to valuable recommendations to enhance patient safety and mitigate potential errors.

Per the TG‐100 guideline, during the FMEA analysis, we did not assume routine quality assurances were in place. The analysis pinpointed the most critical steps in the radiotherapy workflow at our institution that pose significant hazards. The existing literature indicates that an RPN value exceeding 125 is deemed unacceptable for any risk.^19^ Other FMEA studies suggest that RPN scores exceeding 150 warrant the implementation of safety interventions.^20^ Despite the valuable insights offered by these publications, there remains an absence of a universally accepted standard for identifying the threshold at which an RPN score requires attention. In contrast to prior studies that solely relied on either RPN or a risk matrix to assess the risk levels, our novel approach of incorporating a risk matrix alongside RPN considerations demonstrated that RPNs significantly lower than the thresholds proposed in the literature may still indicate a high level of risk and require specific attention.^21^, ^22^, ^23^ Conversely, certain steps with low‐risk levels might yield high RPN values due to their high occurrence rate. The analysis showed that human failures, such as inexperience, inattention, and a lack of thorough checks, are identified as the primary potential causes of these high‐risk failure modes. In their study, Klüter et al. found that catastrophic machine errors were consistently ranked lower than human errors in the context of online ART.^21^ In our study, contouring errors were notably among the highest RPN failure modes, mirroring trends observed in similar research. Specifically, the study by Noel et al. reported that 75% of the increased RPN values in adaptive radiotherapy were attributed to failures in segmentation and treatment planning.^24^ Additionally, the research conducted by Bin et al. is particularly relevant to our findings. In their analysis of adaptive radiotherapy, they identified “Incorrect target/structure delineation and construction” and “Poor plan optimization and/or incorrect dose computation” as the fourth and fifth highest‐ranking failure modes.^25^ Nishioka et al. ranked the wrong prescription as the highest RPN failure mode, as was the case in our study.^6^ These parallels underscore the recurring challenge of target delineation and plan optimization in ART processes. The potential effects of these failures range from tumor overdose or underdose to normal tissue complication. A noteworthy finding is that all high‐risk failure modes fell within either the top 20% of RPNs or the top 20% of severe failure modes.

To address the identified failure modes and improve patient safety, continuous education and training programs with various professional groups were held to enhance the skills and knowledge of personnel involved in the process and reduce the associated risks. An incident report and learning system also plays a crucial role in data collection and workflow optimization, coupled with periodic team huddles. From this optimal workflow, we extended our analysis to include a TDABC analysis. While previous studies have examined comparisons of various treatment modalities in radiation oncology, limited research has been conducted on the comparative cost analysis of CTgRT and MRgRT.^26^, ^27^, ^28^, ^29^, ^30^ In the present study, we employed TDABC to evaluate the costs associated with MRgRT (with online re‐planning) and compare them with conventional CTgRT (without online re‐planning) for administering a five‐fraction SBRT treatment course to patients with liver, lung, and pancreatic cancers. Our analysis of forty patient cases considered the various steps involved in CDVC for each treatment method and estimated the costs of resources supplied during the treatments. Parikh et al. conducted a similar, rigorous TDABC study to compare the costs associated with MRgRT utilizing MRIdian and CTgRT using TrueBeam for administering a five‐fraction SBRT to patients with localized unresectable hepatocellular carcinoma.^31^ In their study, MRgRT was found to have a comparable cost to CTgRT. However, our research revealed that MRgRT is 123% more expensive than CTgRT. This significant cost discrepancy primarily arises from their elevated personnel and material costs associated with CTgRT on the simulation day, as well as the higher equipment costs of CTgRT on the treatment day.

Notwithstanding the greater cost relative to CTgRT, MRgRT presents numerous remarkable advantages, encompassing improved target localization and real‐time tracking‐and‐gating, enhanced normal tissue sparing, daily adaptive re‐planning, and promising avenues such as biology‐guided RT through functional MR imaging.^17^, ^18^, ^32^, ^33^ Furthermore, MRgRT's integration of AI‐driven technologies holds the potential for significantly improving and further streamlining the workflow, rendering it a compelling contender in the field of radiotherapy. Due to the unprecedented accuracy and margin reductions afforded by the technology, further hypofractionation below five fractions may be possible in the future. This would further reduce the overall cost of the MRgRT. So, it may not be entirely impossible that the costs become comparable to CTgRT.

Our current study specifically focused on calculating the costs associated with MRgRT and CTgRT workflows, thereby highlighting the economic aspects of these treatment modalities. Given the varying motion sensitivity of different organs and their respective responses to MRgRT and CTgRT, we recognize that the cost of the workflow alone cannot be the sole deciding factor in choosing the treatment modality. In future studies focusing on cost‐effectiveness, the comprehensive cost data from our research will play a crucial role. By integrating our findings with organ‐specific analyses, future research can extend beyond mere cost calculation to include Quality‐Adjusted Life Years (QALY) and the potential cost implications of complications arising from tissue overdoses. This multifaceted approach will provide a more complete understanding of the economic and clinical viability of MRgRT and CTgRT, especially for treatment sites prone to significant motion. Consequently, our study sets a foundational groundwork for these comprehensive cost‐effectiveness analyses, crucial for informed decision‐making in radiotherapy.

## 5 CONCLUSION

MRgRT offers unique advantages in delivering precise radiation therapy with real‐time MR imaging, target tracking‐and‐gating, and flexible adaptive replanning capabilities. However, careful consideration of costs is essential for widespread adoption and sustainability. Technologies such as auto‐segmentation, synthetic CT, increased radiation dose rates, MLC tracking, and VMAT show promise in reducing MRgRT costs. Our analysis revealed which technologies are more effective for cost savings and which may not yield significant benefits. This work contributes valuable data and insights to the domain of efficient and cost‐effective healthcare, particularly in light of the recent bankruptcy of the MR‐Linac vendor, ViewRay. This development highlights the critical need for thorough cost analysis and efficient utilization of advanced medical technologies like MR‐Linac systems. Our findings provide key insights that can inform future strategies for healthcare providers and technology developers, ensuring sustainable and financially viable use of such advanced systems in clinical practice.

## AUTHOR CONTRIBUTIONS

Mojtaba Behzadipour: Conceptualization, Acquisition, analysis and interpretation of data, Methodology, Validation, Visualization, Drafting the work, Final approval of the version. Jatinder Palta: Conceptualization, Resources, Interpretation of data, Methodology, Validation, Revising the draft, Final approval of the version. Tianjun Ma: Conceptualization, Interpretation of data, Methodology, Validation, Revising the draft, Final approval of the version. Lulin Yuan: Conceptualization, Acquisition, Interpretation of data, Methodology, Validation, Revising the draft, Final approval of the version. Siyong Kim: Conceptualization, Interpretation of data, Resources, Methodology, Validation, Revising the draft, Final approval of the version. Suzanne Kirby: Acquisition, analysis, Interpretation of data, Validation, Visualization, Revising the draft, Final approval of the version. Laurel Torkelson: Acquisition, analysis, Interpretation of data, Validation, Visualization, Revising the draft, Final approval of the version. James Baker: Acquisition, analysis, Interpretation of data, Validation, Visualization, Revising the draft, Final approval of the version. Tammy Koenig: Acquisition, analysis, Interpretation of data, Validation, Visualization, Revising the draft, Final approval of the version. Mateb Al Khalifa: Conceptualization, Acquisition, Interpretation of data, Methodology, Validation, Revising the draft, Final approval of the version. Robert Hawranko: Conceptualization, Acquisition, Interpretation of data, Methodology, Validation, Revising the draft, Final approval of the version. Dylan Richeson: Conceptualization, Acquisition, Interpretation of data, Methodology, Validation, Revising the draft, Final approval of the version. Emma Fields: Conceptualization, Acquisition, Interpretation of data, Methodology, Validation, Revising the draft, Final approval of the version. Elisabeth Weiss: Conceptualization, Acquisition, Interpretation of data, Methodology, Validation, Revising the draft, Final approval of the version. William Y. Song: Conceptualization, Resources, Acquisition, Interpretation of data, Methodology, Validation, Drafting the work, Revising the draft, Final approval of the version.

## CONFLICT OF INTEREST STATEMENT

William Y. Song: Research funding and honoraria.

## References

[1] Tyagi N , Liang J , Burleson S , et al. Feasibility of ablative stereotactic body radiation therapy of pancreas cancer patients on a 1.5 Tesla magnetic resonance‐linac system using abdominal compression. Phys Imaging Radiat Oncol. 2021;19:53‐59. 34307919 10.1016/j.phro.2021.07.006 PMC8295846

[2] Corradini S , Alongi F , Andratschke N , et al. MR‐guidance in clinical reality: current treatment challenges and future perspectives. Radiat Oncol. 2019;14(1):1‐12. 31167658 10.1186/s13014-019-1308-y PMC6551911

[3] Chandarana H , Wang H , Tijssen R , Das IJ . Emerging role of MRI in radiation therapy. J Magn Reson Imaging. 2018;48(6):1468‐1478. 30194794 10.1002/jmri.26271 PMC6986460

[4] Maziero D , Straza MW , Ford JC , et al. MR‐guided radiotherapy for brain and spine tumors. Front Oncol. 2021;11:626100. 33763361 10.3389/fonc.2021.626100 PMC7982530

[5] Otazo R , Lambin P , Pignol J‐P , et al. MRI‐guided radiation therapy: an emerging paradigm in adaptive radiation oncology. Radiology. 2021;298(2):248‐260. 33350894 10.1148/radiol.2020202747 PMC7924409

[6] Nishioka S , Okamoto H , Chiba T , et al. Identifying risk characteristics using failure mode and effect analysis for risk management in online magnetic resonance‐guided adaptive radiation therapy. Phys Imaging Radiat Oncol. 2022;23:1‐7. 35712526 10.1016/j.phro.2022.06.002 PMC9194450

[7] Huq MS , Fraass BA , Dunscombe PB , et al. The report of Task Group 100 of the AAPM: application of risk analysis methods to radiation therapy quality management. Med Phys. 2016;43(7):4209‐4262. 27370140 10.1118/1.4947547 PMC4985013

[8] Kaplan RS , Anderson SR . Time‐driven activity‐based costing. Available at SSRN 485443. 2003. 15559451

[9] Klüter S . Technical design and concept of a 0.35 T MR‐Linac. Clin Transl Radiat Oncol. 2019;18:98‐101. 31341983 10.1016/j.ctro.2019.04.007 PMC6630153

[10] Liney GP , Whelan B , Oborn B , Barton M , Keall P . MRI‐linear accelerator radiotherapy systems. Clin Oncol. 2018;30(11):686‐691. 10.1016/j.clon.2018.08.003 30195605

[11] Sohn J , Guy C , Datsang R , Kim S . Touchless compression using shallow kinetics induced by metronome (SKIM). Int J Radiat Oncol Biol Phys. 2021;111(3):S48.

[12] Güngör G , Serbez İ , Temur B , et al. Time analysis of online adaptive magnetic resonance–guided radiation therapy workflow according to anatomical sites. Pract Radiat Oncol. 2021;11(1):e11‐e21. doi:10.1016/j.prro.2020.07.003 32739438

[13] Cardenas CE , Yang J , Anderson BM , Court LE , Brock KB . Advances in auto‐segmentation. Semin Radiat Oncol. 2019;29(3):185‐197. doi:10.1016/j.semradonc.2019.02.001 31027636

[14] Harrison K , Pullen H , Welsh C , Oktay O , Alvarez‐Valle J , Jena R . Machine learning for auto‐segmentation in radiotherapy planning. Clin Oncol. 2022;34(2):74‐88. doi:10.1016/j.clon.2021.12.003 34996682

[15] Lim JY , Leech M . Use of auto‐segmentation in the delineation of target volumes and organs at risk in head and neck. Acta Oncol (Madr). 2016;55(7):799‐806. doi:10.3109/0284186X.2016.1173723 27248772

[16] Hawranko R , Sohn JJ , Neiderer K , et al. Investigation of isotoxic dose escalation and plan quality with TDABC analysis on a 0.35 T MR‐Linac (MRL) system in ablative 5‐fraction stereotactic magnetic resonance‐guided radiation therapy (MRgRT) for primary pancreatic cancer. J Clin Med. 2022;11(9):2584. 35566712 10.3390/jcm11092584 PMC9104241

[17] Jaffray DA , Carlone MC , Milosevic MF , et al. A Facility for Magnetic Resonance–Guided Radiation Therapy. Elsevier; 2014:193‐195. 10.1016/j.semradonc.2014.02.012 24931091

[18] Kerkhof EM , Raaymakers BW , van der Heide UA , van de Bunt L , Jürgenliemk‐Schulz IM , Lagendijk JJ . Online MRI guidance for healthy tissue sparing in patients with cervical cancer: an IMRT planning study. Radiother Oncol. 2008;88(2):241‐249. 18490068 10.1016/j.radonc.2008.04.009

[19] Huq MS , Fraass BA , Dunscombe PB , et al. A method for evaluating quality assurance needs in radiation therapy. Int J Radiat Oncol Biol Phys. 2008;71(1):S170‐S173. 18406920 10.1016/j.ijrobp.2007.06.081

[20] Ford EC , Smith K , Terezakis S , et al. A streamlined failure mode and effects analysis. Med Phys. 2014;41(6Part1):061709. 24877804 10.1118/1.4875687

[21] Klüter S , Schrenk O , Renkamp CK , et al. A practical implementation of risk management for the clinical introduction of online adaptive magnetic resonance‐guided radiotherapy. Phys Imaging Radiat Oncol. 2021;17:53‐57. 33898779 10.1016/j.phro.2020.12.005 PMC8058032

[22] Rusu I , Thomas TO , Roeske JC , Mescioglu I , Melian E , Surucu M . Failure mode and effects analysis of linac‐based liver stereotactic body radiotherapy. Med Phys. 2020;47(3):937‐947. 31837024 10.1002/mp.13965

[23] Liang J , Scripes PG , Tyagi N , et al. Risk analysis of the Unity 1.5 T MR‐Linac adapt‐to‐position workflow. J Appl Clin Med Phys. 2023;24(3):e13850. 36411990 10.1002/acm2.13850 PMC10018675

[24] Noel CE , Santanam L , Parikh PJ , Mutic S . Process‐based quality management for clinical implementation of adaptive radiotherapy. Med Phys. 2014;41(8Part1):081717. 25086527 10.1118/1.4890589 PMC4119199

[25] Cai B , Green OL , Kashani R , Rodriguez VL , Mutic S , Yang D . A practical implementation of physics quality assurance for photon adaptive radiotherapy. Zeitschrift für Medizinische Physik. 2018;28(3):211‐223. 29550014 10.1016/j.zemedi.2018.02.002

[26] Beriwal S , Chino J . Time‐driven activity‐based costing in oncology: a step in the right direction. Int J Radiat Oncol Biol Phys. 2018;100(1):95‐96. 29254783 10.1016/j.ijrobp.2017.10.017

[27] Dutta SW , Bauer‐Nilsen K , Sanders JC , et al. Time‐driven activity‐based cost comparison of prostate cancer brachytherapy and intensity‐modulated radiation therapy. Brachytherapy. 2018;17(3):556‐563. 29519605 10.1016/j.brachy.2018.01.013

[28] Laviana AA , Ilg AM , Veruttipong D , et al. Utilizing time‐driven activity‐based costing to understand the short‐and long‐term costs of treating localized, low‐risk prostate cancer. Cancer. 2016;122(3):447‐455. 26524087 10.1002/cncr.29743

[29] Pezzi TA , Ning MS , Thaker NG , et al. Evaluating single‐institution resource costs of consolidative radiotherapy for oligometastatic non‐small cell lung cancer using time‐driven activity‐based costing. Clin Transl Radiat Oncol. 2020;23:80‐84. 32529054 10.1016/j.ctro.2020.05.007 PMC7283089

[30] Schutzer ME , Arthur DW , Anscher MS . Time‐driven activity‐based costing: a comparative cost analysis of whole‐breast radiotherapy versus balloon‐based brachytherapy in the management of early‐stage breast cancer. J Oncol Pract. 2016;12(5):e584‐e593. 27006360 10.1200/JOP.2015.008441

[31] Parikh NR , Lee PP , Raman SS , et al. Time‐driven activity‐based costing comparison of CT‐guided versus MR‐guided SBRT. JCO Oncol Pract. 2020;16(11):e1378‐e1385. 32539652 10.1200/JOP.19.00605

[32] Botman R , Tetar S , Palacios M , Slotman B , Lagerwaard F , Bruynzeel A . The clinical introduction of MR‐guided radiation therapy from a RTT perspective. Clin Transl Radiat Oncol. 2019;18:140‐145. 31341990 10.1016/j.ctro.2019.04.019 PMC6630155

[33] van Timmeren JE , Chamberlain M , Krayenbuehl J , et al. Treatment plan quality during online adaptive re‐planning. Radiat Oncol. 2020;15(1):1‐11. 10.1186/s13014-020-01641-0 PMC7441614 32825848

